# Indications for involuntary hospitalization for refusal of treatment in severe anorexia nervosa: a survey of physicians and mental health care review board members in Japan

**DOI:** 10.1186/s40337-022-00703-w

**Published:** 2022-11-21

**Authors:** Yoshiyuki Takimoto

**Affiliations:** 1 Department of Biomedical Ethics, Faculty of Medicine, The University of Tokyo, Tokyo, Japan; 2 Department of Psychosomatic Medicine and Stress Science, Faculty of Medicine, The University of Tokyo, Tokyo, Japan

**Keywords:** Anorexia nervosa, Involuntary hospitalization, Ethics

## Abstract

**Background:**

When a patient with anorexia nervosa refuses treatment despite a physically critical condition, the therapist considers involuntary inpatient treatment under the Mental Health Law. However, ethical and practical problems arise from its application. In this study, a survey of treatment providers for eating disorders and psychiatric review board members was conducted regarding indications for involuntary hospitalization under the Mental Health Act for refusal of treatment for anorexia nervosa.

**Methods:**

A survey of 212 physicians affiliated with the Japanese Society for Eating Disorders and 180 members of Mental Health Care Review Boards across Japan was conducted using six vignette cases of patients with anorexia nervosa refusing treatment.

**Results:**

Regardless of the duration of illness or age of the patient, few physicians chose compulsory hospitalization with or without the consent of the family, while the largest number of physicians chose hospitalization for medical care and protection when there was family consent. Among committee members, only hospitalization for medical care and protection was determined to be appropriate when there was family consent. Both hospitalization for medical care and protection, and compulsory hospitalization were deemed appropriate in the absence of family consent. Committee members who adjudged refusal of treatment for anorexia nervosa as self-injurious behavior suggested that compulsory hospitalization was indicated.

**Conclusions:**

When a patient with life-threatening anorexia nervosa refuses inpatient treatment, hospitalization for medical care and protection is actively chosen if the patient’s family consents. Mental Health Care Review Board members considered this acceptable. However, if the family does not consent, the physicians did not choose compulsory hospitalization, and the psychiatric review board was divided on this. Consensus was not achieved in this regard.

**Supplementary Information:**

The online version contains supplementary material available at 10.1186/s40337-022-00703-w.

## Plain English summary

When a patient with anorexia nervosa refuses treatment despite a physically critical condition, it raises an ethical dilemma. If the patient in a life-threatening condition refuses treatment, physicians consider involuntary hospitalization for treatment as per the law. To date, there is no uniform decision in this regard. Therefore, in this study, a survey of treatment providers for eating disorders and psychiatric review board members was conducted regarding indications for involuntary hospitalization under the Mental Health Act for refusal of treatment for anorexia nervosa. When family members consented to treatment, the physicians considered hospitalization for medical care and protection, and the members also agreed that this was indicated. However, when the family did not consent, the physicians did not choose compulsory hospitalization, and the members were divided on the indications for compulsory hospitalization. Therefore, the handling of cases wherein patients with anorexia nervosa refuse inpatient treatment despite a life-threatening condition, in addition to their families not providing consent for treatment, will be extremely difficult.

**Supplementary Information:**

The online version contains supplementary material available at 10.1186/s40337-022-00703-w.

## Background

Management of patients with anorexia nervosa (AN) is difficult if the patient refuses treatment. Specifically, when a patient is in a physically critical condition owing to undernutrition and refuses treatment due to the psychopathology of AN, the treatment provider is faced with the ethically difficult decision of whether to force treatment in order to protect the patient or whether to give priority to the patient’s preference. The Act on Mental Health and Welfare for the Mentally Disabled (MHA) ordained four types of hospitalization for mental illness as follows: “voluntary hospitalization” (Article 22-3 of MHA), whereby the patient himself/herself consents to hospitalization; “hospitalization for medical care and protection (HMCP)” (Article 33 of MHA), whereby one of the family members consents, “compulsory hospitalization (CH)” (Article 29 of MHA), which involves hospitalization under the authority of the prefectural governor; and “emergency hospitalization” (Article 34-4 of MHA), for emergency evacuation. (“Act on Mental Health and Welfare for the Mentally Disabled,” May 1, 1950). In Japan, two options exist when a physician chooses to provide involuntary treatment. Of the aforementioned types of hospitalizations under MHA, HMCP and CH are subject to consideration as a response to the case of a patient with AN who refuses treatment despite being in a high-risk physical condition [1].

HMCP is for patients who require hospitalization and are not in a condition to be voluntarily hospitalized, although there is no risk of self-injury or other harm, and CH is for patients who are at risk of self-injury or other harm if they are not hospitalized. There is considerable ethical debate regarding the treatment of patients with AN. In addition to the ethical considerations, practicality must be addressed when considering the actual legality of involuntary inpatient treatment.

First, both HMCP and CH assume that the patient cannot be hospitalized on his/her own accord, and that the patient has lost the mental capacity regarding voluntary hospitalization. However, the existence or non-existence of the mental capacity for AN is controversial [2, 3]. Second, the mental disorders covered by HMCP and CH are psychiatric disorders (e.g., schizophrenia and major depressive disorder) with obvious psychiatric symptoms. One perspective is that AN is not a psychiatric condition, but an understandable behavior to meet specific needs, for example being skinny [4]; therefore, AN, which has limited psychiatric symptoms (i.e., mainly the fear of obesity), may not be a mental disorder covered by HMCP and CH. Third, the interpretation of the fear of self-injury or other harm as a requirement for CH needs to be evaluated. Although the refusal to eat can cause a physically critical condition in patients with AN, whether this indeed constitutes self-injurious behavior needs confirmation.

To ensure that the management of a mental disorder is balanced against a person’s human rights, the Mental Health Care Review Board (MHCRB) was established under the MHA. This body professionally and independently examines the treatment of persons with a mental disorder admitted to psychiatric hospitals [5]. The Board specifically examines the following: (1) hospitalization notifications submitted by hospital administrators to the government for persons admitted for HMCP and periodic medical condition reports for persons admitted for HMCP and CH to determine whether their hospitalization is necessary and whether their treatment is appropriate; (2) whether the hospitalization is necessary and whether the treatment for those who are hospitalized in psychiatric hospitals is appropriate in response to requests for discharge or improvement of the treatment from patients or their families [5].

Ethical and legal arguments surround the compulsory treatment of patients with AN who refuse treatment [6, 7]. In contrast, physicians may be in situation where they have no choice but to admit patients under HMCP or CH to save their lives in response to refusal of treatment for AN [8–10]. The number of patients with AN who are hospitalized for HMCP and CH is not clear, as is the application of involuntary hospitalization based on MHA for the refusal of treatment for AN. Furthermore, there have been no reports comparing the perspectives of indications for involuntary hospitalization of patients with AN between physicians and MHCRB reviewers. Therefore, the purpose of this study was to document the frequency of MHCRB decisions regarding HMCP and CH for the treatment of AN and to clarify their decision-making process. A questionnaire survey was completed by MHCRB members. Additionally, the decision-making process of physicians specializing in treatment of eating disorders with regards to HMCP and CH were also investigated.

## Methods

An assessment of MHCRB activity in the past year, as well as a survey of all MHCRB members and a survey of all the physicians affiliated to the Japan Society for Eating Disorders were conducted.

### Ethics approval and consent to participate

This research was approved by the Ethics Committee of the Faculty of Medicine, the University of Tokyo (No. 10833-(1)). The questionnaires were anonymous, and written informed consent was obtained. All participants provided consent to have their data published.

### Case vignettes

Six fictitious vignettes were used in the study, each comprising a combination of two different patient conditions (cases A, B, and C) and two different reactions of the patients’ families. The respondents were asked whether they would choose compulsory inpatient treatment or not (see Additional file 1). Case A (a minor) and Case B (an adult) had acute anorexia nervosa and Case C was a person with chronic and severe anorexia nervosa.

In order to determine how a physician would act in the situation, the physicians were asked regarding their choice among the possible options of CH, HMCP, persuasion for voluntary hospitalization, and others. Members of the Mental Health Care Review Board were asked to respond to the question regarding their judgment on whether CH or HMCP was legally indicated.

### Survey to treatment providers

An anonymous self-administered questionnaire survey including six vignettes was delivered by mail to 212 members of the Japanese Society for Eating Disorders.

### Survey to the mental health care review board

Written requests for cooperation in the survey were sent to 67 MHCRB in all 47 prefectures to determine the current status of the review, which was assessed by the following factors: number of MHCRBs, number of reviewers, number of admissions for treatment purposes in 1 year, number of admissions for treatment purposes in 1 year for AN, number of admissions for treatment purposes in 1 year for HMCP and CH, number of HMCP and CH for treatment purposes for AN in 1 year, number of discharge claims in 1 year, number of hospitalizations without legal indication or transfer of hospitalization status in 1 year, number of discharge claims for AN in 1 year, and the approved number of hospitalizations without legal indication or change of hospitalization type among AN discharge claims in 1 year. In addition, the review board asked the committee members to complete the questionnaire consisting of the same six vignettes that were sent to physicians from the Japanese Society for Eating Disorders and requested anonymous responses. Furthermore, MHCRB members were asked whether refusing treatment of AN constitutes self-harm as defined in the MHA, whether AN constitutes a mental illness as defined in the MHA, and whether a patient with AN who refuses treatment despite a life-threatening situation has the capacity to make a decision.

### Statistical analysis

The χ^2^ test was used to examine the differences in the responses of the care providers and MHCRB members in cases A, B, and C. If the χ^2^ test result was significant, χ^2^ test or Fisher’s exact method was used to analyze the differences in the responses between two cases, and Bonferroni’s correction was applied. Further, *p* < 0.01 was considered statistically significant. A two-item logistical analysis was conducted to identify factors influencing decision of the committee board members on whether a patient required involuntary hospitalization for either HMCP or CH. The selected explanatory factors were the presence or absence of the patient’s decision-making capacity, whether or not the patient had a mental disorder, and whether or not the patient’s behavior indicated self-harm. Variables were analyzed using forced entry. All analyses were two-tailed, and a *p*-value < 0.05 was considered statistically significant.

## Results

### Results for treatment providers of eating disorders

#### Characteristics of physicians

Fifty-five valid responses were obtained from physicians in Japan who specialize in treating eating disorders (25.9% response rate). The physicians included 21 psychosomatic physicians, 24 psychiatrists, and 10 adolescent medicine physicians. Psychosomatic physicians are trained in internal medicine with additional psychiatric-psychosomatic training. Both psychosomatic physicians and psychiatrists treat eating disorders in Japan. Most physicians had 10 to 19 years of experience, while some had more than 30 years of experience. Most physicians treated 50 to 99 patients in a year, while some treated 150 to 199 patients in a year.

#### Physician attitude to treatment refusal (n = 55) (Table 1)

**Table 1:** Attitudes of physicians towards treatment refusal by patients with severe anorexia nervosa

Case	Compulsory hospitalization	Hospitalization for medical care and protection	Voluntary hospitalization	Other outpatient treatment
Case A 15 yrs (n = 55)				
FW +	0 (0%)	32 (58.2%)	20 (36.4%)	3 (5.5%)
FW −	1 (1.8%)	26 (47.2%)	20 (36.4%)	8 (14.5%)
Case B 20 yrs (n = 55)				
FW +	0 (0%)	31 (56.4%)	19 (34.5%)	5 (9.5%)
FW −	1 (1.8%)	29 (52.7%)	15 (27.3%)	10 (18.2%)
Case C 40 yrs (n = 55)				
FW +	0 (0%)	36 (65.4%)	17 (30.9%)	2 (9.5%)
FW −	0 (0%)	24 (43.6%)	14 (25.4%)	17 (30.9%)

*CH* Compulsory hospitalization, *HMCP * Hospitalization for medical care and protection, *FW*+  Family members wished the patient to be treated. *FW*−  Family members did not wish the patient to be treated

Case A: The patient with AN was 15-years-old with a 6-months disease duration and refused treatment for a life-threatening condition. If the family consented that the patient should be treated, most respondents (58%) opted for HMCP. Even if the family did not consent to treatment for the patient, 47% physicians chose HMCP. Only 2% of the respondents selected CH.

Case B: The patient with AN was 20-years-old with a 6-months disease duration and refused treatment for a life-threatening condition. If the family consented that the patient should be treated, most respondents (56%) chose HMCP. When the family did not consent to treatment for the patient, 53% physicians chose HMCP; however, only 2% respondents selected CH.

Case C: The patient with AN was 40-years-old with a 24-year disease duration, multiple life-threatening events, and refused treatment for a life-threatening condition. If the family consented to treatment, most respondents (65%) chose HMPC. Even when the family did not consent for the patient to be treated, 44% physicians chose HMCP; no one selected CH.

There were no significant differences in response trends among cases A, B, and C where the family members consented for the patient to be treated and where the family members did not consent for the patient to be treated (*p* = 0.95, χ^2^ = 7.18, df = 4 and *p* = 0.794, χ^2^ = 3.12, df = 6, respectively).

### Results for the mental health care review board

Responses were received from 23 (34.3%) of the 67 MHCRB. Questionnaires were also individually distributed to 180 members of the MHCRB, and responses were obtained from 77 members (42.8%).

#### Organization of the mental health care review board (n = 23)

The median number of MHCRBs was 3 (min: 1, max: 8) and the median number of reviewers per MHCRB was 18 (min: 10, max: 40).

#### Characteristics of reviewers (n = 77)

Of the total reviewers, 59% had an academic background in psychiatry (n = 45), 9% had an academic background in law (n = 7), 27% had other academic backgrounds (n = 21), and 5% provided no response (n = 4).

#### Review by mental health care review board for 1 year

The median number of CH during the year (from n = 22 responding facilities) was 19 (min 0, max 97), of which none were for patients with AN (from n = 19 responding facilities). The median number of HMCP (from n = 22 responding facilities) during the year was 2259.5 (min: 968, max: 8947), of which 7 (min: 0, max: 25) were for patients with AN (from n = 18 responding facilities). The median number of discharge claims (from n = 23 responding facilities) was 27 (min: 5, max: 344) and the median number of admissions with unjustified admission or transition of admission status (from n = 23 responding facilities) was 1 (min: 0, max: 16). Of these, the median number of discharge requests by patients with AN (from n = 22 responding facilities) was 0 (min: 0, max: 1), and the median number of cases of inadvertent hospitalization or transition of admission status (n = 19 responding facilities) was 0 (min: 0, max: 1).

#### Mental health care review board decisions of indication for involuntary hospitalization for refusal of treatment by AN (n = 77) (Table 2)

**Table 2:** Psychiatric review board members' judgement of indication for involuntary hospitalization after patients’ treatment refusal

Case	Both CH and HMCP	Compulsory hospitalization	Hospitalization for medical care and protection	No indication for compulsory hospitalization
Case A 15 yrs (n = 77)				
FW +	21 (27.3%)	0 (0%)	53 (68.8%)	33 (3.9%)
FW −	6 (7.8%)	34 (44.2%)	3 (3.9%)	34 (44.2%)
Case B 20 yrs (n = 77)				
FW +	21 (27.3%)	1 (1.3%)	52 (67.5%)	3(3.9%)
FW −	7 (9.1%)	30 (39.0%)	6 (7.8%)	34 (44.2%)
Case C 40 yrs (n = 77)				
FW +	19 (24.7%)	0 (0%)	53 (68.8%)	5 (6.5%)
FW −	5 (6.5%)	27 (35.1%)	3 (3.9%)	42 (54.5%)

*CH* Compulsory hospitalization, *HMCP* Hospitalization for medical care and protection, *FW*+  Family members wished the patient to be treated. *FW*− Family members did not wish the patient to be treated

For Case A, if the family members consented to treatment for the patient, most respondents (69%) decided that only HMCP was indicated. In cases where the family did not consent to treatment for the patient, 44% answered that neither CH nor HMCP would be indicated, while another 44% answered that only CH would be indicated.

For Case B if the family members consented to treatment for the patient, most respondents (67%) decided that only HMCP was indicated. In cases where the family did not consent to treatment for the patient, 44% answered that neither CH nor HMCP would be indicated, and 39% answered that only CH would be indicated.

For Case C, if the family members consented to treatment for the patient, most respondents (69%) decided that only HMCP was indicated. In cases where the family did not consent to treatment for the patient, 55% answered that neither CH nor HMCP would be indicated.

There were no significant differences in response trends among cases A, B, and C where the family members wished the patient to be treated and where the family members did not wish the patient to be treated (*p* = 0.83, χ^2^ = 2.87, df = 6 and *p* = 0.70, χ^2^ = 3.81, df = 6, respectively).

#### Applicable to mental disability (n = 73)

When asked whether AN applies to mental disorders treated under the Mental Health and Welfare Law, 69 (94%) MHCRB members responded that this condition applies to mental disorders, and 4 (6%) responded that it does not.

#### Applicable to self-injury behavior

When asked whether refusal of nutritional treatment constituted self-injurious behavior as treated under the Mental Health and Welfare Law, 46 (62%) responded that it constituted self-injurious behavior, while 28 (38%) said that it did not.

#### Mental capacity

The assessment of mental capacity for the presented case (see Additional file 2) was “full mental capacity” for 8 (11%), “partially impaired but has good mental capacity” for 40 (55%), and “lack of mental capacity” for 25 (34%) respondents. Further, the 25 respondents who indicated “lack of mental capacity” were asked about the reasons for their response. Two (8%) respondents stated decreased level of consciousness due to undernourishment, 22 (88%) stated decreased mental capacity due to AN psychopathology (desire to be thin, fear of obesity), and one (4%) had other reasons. When 48 respondents, who assessed the presence of mental capacity, were asked to confirm whether the patient’s self-decision to refuse treatment should be respected, eight (20%) answered that it should be respected and 40 (80%) replied that it should not. When the same question was asked of physicians treating patients with AN (n = 53), six respondents (11%) indicated that the patients had full mental capacity, 35 (66%) indicated that “partial impairment but good mental capacity,” and 12 (23%) indicated that the patients lacked mental capacity. No significant differences were found between the reviewers and the physicians with respect to their assessment of mental capacity (*p* = 0.16, χ^2^ = 1.99, df = 1).

#### Factors influencing the decision to indicate involuntary hospitalization

When the family members consented for the patient to be treated, neither the presence of mental capacity applicable to mental disorders nor to self-injury had a significant effect on their decision to support involuntary inpatient treatment (Table 3). If the family did not consent for the patient to be treated, in cases where the MHCRB members considered AN behaviors to be self-injurious, the decision to hospitalize involuntarily was 34.7 times higher in Case A (odds ratio: 34.71 [7.89–152.72] p < 0.001), 34.7 times higher in Case B (odds ratio: 34.71 [7.89–152.72] *p* < 0.001), and 14.9 times more likely in Case C (odds ratio 14.91 [3.65–60.89] *p* < 0.001). In these instances, the self-injurious nature of the behavior was considered to be an indication for involuntary hospitalization.

**Table 3:** Factors influencing involuntary hospitalization indication after treatment refusal by patients with severe anorexia nervosa

Case	Mental capacity (− or +) Ref −		Qualifies as mental disability (No or Yes) Ref No		Qualifies as self − injury (No or Yes) Ref No		Hosmer and Lemeshow
	Odds (CI)	*p*-value	Odds (CI)	*p*-value	Odds (CI)	*p*-value	*p*-value
Case A15 yrs FW +	5.6 $\times$ 10^−8^	0.99	4.75 (0.29 − 78.74)	0.28	8.48 $\times$ 10^7^	0.99	1.000
Case A 15 yrs FW −	1.03 (0.27 − 3.98)	0.97	1.40 (0.06 − 33.77)	0.84	**34.71** (7.89 − 152.72)	** < 0.001**	0.88
Case B 20 yrs FW +	5.6 $\times$ 10^−8^	0.99	4.75 (0.29 − 78.74)	0.28	8.48 $\times$ 10^7^	0.99	1.000
Case B 20 yrs FW −	1.03 (0.27 − 3.98)	0.97	1.40 (0.06 − 33.77)	0.84	**34.71** (7.89 − 152.72)	** < 0 .001**	0.88
Case C 40 yrs FW +	1.50 (0.11 − 21.58)	0.77	5.26 (0.34 − 81.61)	0.24	5.15 (0.39 − 66.46)	0.21	0.61
Case C 40 yrs FW −	1.41 (0.45 − 4.41)	0.56	0.24 (0.02 − 2.77)	0.25	**14.91** (3.65 − 60.89)	** < 0.001**	0.67

Bolds indicate statistical significance

*CI* confidence interval, *FW*+ Family members wished the patient to be treated, *FW*− Family members did not wish the patient to be treated

## Discussion

To the best of the author’s knowledge, this is the first study about the attitudes of MHCRB members, who review involuntary hospitalization under the law, towards the indications for involuntary hospitalization of patients with AN that refuse treatment despite a life threatening condition. In the event that a patient refused inpatient treatment despite a possible life threatening condition, more than half of the physicians chose HMCP if the family consented for the patient to be treated. Almost all MHCRB members also judged that HMCP was indicated, and their views were consistent. This trend did not differ by patient age or the duration of illness.

Treatments are implemented if they are medically effective regarding the principle of beneficence and if the patient’s consent is obtained based on the principle of respect for autonomy. Many legal experts recommend imposing legal interventions to require treatment only in those situations where the patient is in a critical physical condition and unable to consent to treatment of his/her own accord [11]. However, only about 30% of the physicians and members in this study rated patients with AN as completely lacking in mental capacity. In the current survey, only about 30% of the physicians and review board members rated patients with AN as completely incapable of making decisions. However, approximately 60% of the physicians and review board members reported that the mental capacity of patients with AN was partially impaired. In general, mental capacity is evaluated relative to the treatment action proposed and the reason for which it is intended. In particular, high mental capacity is considered necessary to refuse treatment with low risk and high benefit. Treatment through involuntary hospitalization for patients with AN has been shown to have at least a short-term therapeutic effect for some patients [12]. Therefore, the physicians and board members may believe that patients with AN who refuse treatment, while not completely incapacitated, are unlikely to have sufficient mental capacity to refuse treatment to prevent a life-threatening situation and need family support in their decision-making, and thus they may support medical protective hospitalization. In that case, their ethical attitude is based on the concept of relational autonomy [13], which states that the patient’s relationship with and support of those around them is important for their autonomous existence.

In Japan, family-centeredness based on collectivism, which has a Confucian cultural background [14], are thought to influence the values of physicians and the general public regarding the medical field. In a previous study by Ruhnke et al. [15] comparing Japan and the U.S., it was found that both physicians and patients in Japan tended to prefer family-centered decision-making for refractory cancer notification and end-of-life care treatment. The tendency toward family-centered care in Japan may have encouraged physicians and MHCRB members to emphasize relational autonomy in decision making. As a result, HMCP may have been indicated even in cases B and C, comprising adult patients.

Following HMCP, about one-third of the physicians chose voluntary hospitalization, indicating an attempt to avoid involuntary hospitalization. In fact, the number of HMCP patients with AN per year was only a few per MHRCB, suggesting that HMCP was not implemented very often. This may be because physicians consider voluntary hospitalization preferable, and admitting a patient under HMPC to avoid a life-threatening situation may interfere with the subsequent therapeutic relationship and may not be beneficial to the patient in the long-term. The long-term prognosis of patients after involuntary hospitalization is unknown [16].

If the patient is unable to consent to treatment on his/her own, the treatment plan is legally determined according to the best interests of the patient considering the medical benefit or according to the best interpretation of patient’s will and preferences based on the family’s presumption of patient’s intent. Choosing CH in situations where the family did not provide consent was thought to be based on the best interests of the patient considering the medical benefit [17]. One reason why physicians do not choose CH—when the family has not consented for hospitalization but hospitalization is necessary—is their concern regarding the potential deterioration in the therapeutic relationship with the patient and that with the family, which may increase the difficulty of future patient management. Another reason might be the fear of litigation: even if the choice of CH is not legally problematic, the lawsuit itself would be very stressful for the physicians [18]. While judgment regarding indications for CH was accepted by the board members, about half of them considered that there was no indication for CH. There seems to be a lack of consensus on the appropriateness of CH for patients with AN who refuse inpatient treatment in a life-threatening situation and when their families do not wish for them to be treated. One reason why there is disagreement regarding the indications for CH—in situations when there is a lack of family consent for hospitalization, but where hospitalization is necessary—is that if the patient’s refusal of treatment for AN is not considered to constitute self-injurious behavior, then in turn, CH would not be indicated. Specifically, a patient with AN requiring hospitalization and considered to exhibit self-injurious behavior becomes an indication for CH.

Whether or not to regard the refusal of treatment by a patient with AN as self-injurious influenced the decisions regarding indications for hospitalization by MHCRB members. The reviewers who regarded the refusal of treatment with AN as self-injurious were 34.7, 34.7, and 14.9 times more likely to judge this as an indication for CH for cases A, B, and C, respectively. Whether refusal of treatment by a patient with AN, despite a life-threatening condition, constitutes self-injurious behavior is debatable.

When a patient with AN refuses treatment, involuntary inpatient treatment is ethically and legally supported when treatment is necessary to avert a life-threatening risk and when the treatment is likely to be effective. Therefore, the closer the AN condition is to being terminal, the less likely the treatment will be effective, and therefore involuntary inpatient treatment is not considered to be supportive. However, in the present study, there were no differences in attitudes toward legal involuntary inpatient treatment among the groups of physicians and MHCRB members when comparing cases A, B, and C. When judging case C involving a 40-year-old patient, respondents may have considered that the case was not terminal and should be treatable.

The current study had the following limitations. First, since the survey used vignettes, the rationale behind the choices of the respondents were unknown. More detailed information could be obtained through qualitative research. Second, the vignette did not contain detailed clinical information. This may have led to some variations among respondents regarding the medical judgment of the vignette case. How respondents react to a simplified hypothetical written case may be considerably different to how they might respond when confronted by a real situation, with greater complexities and complications. These very brief vignettes are primarily likely to trigger the respondents’ initial intervention biases. Third, the response rate for this survey was not high. Although the survey is highly representative of Japanese Society for Eating Disorders members and Mental Health Care Review Boards in all 47 prefectures, generalizability is limited based on the response rate. Fourth, since the order in which the vignettes were presented was fixed, it cannot be ruled out that this may have created a certain mindset in the respondents, resulting in later responses reflecting the influence from the previous responses.

## Conclusion

In this study, when family members consented to treatment, the physicians considered HMCP, and the members also agreed that HMCP was indicated. However, when the family did not consent, the physicians did not choose CH, and the members were divided on the indications for CH. Therefore, handling of cases wherein patients with AN refuse inpatient treatment despite their life-threatening condition, and in addition, their families do not give their consent for treatment, will be extremely difficult. In the future, it may be necessary to conduct an awareness survey using vignette cases wherein further detailed clinical information has been added, as well as interview surveys to clarify the thoughts and values of physicians and members regarding their attitudes. Furthermore, procedural justice and fairness [19] views this heterogeneity in physicians’ attitudes toward such cases to be undesirable. If possible, it is desirable that expert guidelines are developed to reduce the lack of consensus in clinical practice. The results of this survey may serve as foundational information to achieve agreement.

## Supplementary Information

**Additional file 1**. Presentation of the cases and questions to the respondents.

**Additional file 2**. Case to inquire about the presence or absence of mental capacity.

## Data Availability

The data that support the findings of this study are available from the corresponding author on reasonable request.

## Abbreviations

AN: Anorexia nervosa
CH: Compulsory hospitalization
HMPC: Hospitalization for medical care and protection
MHA: The act on mental health and welfare for the mentally disabled
MHCRB: Mental health care review board

## References

[1] Kondo T, Takaoka K, Ikawa N, Niwa N (2004). Law relating to compulsory treatment of patients with anorexia nervosa. Int Med J.

[2] Fernández-Hernández JL, Herranz-Hernández P, Segovia-Torres L (2021). Refusal of treatment for anorexia nervosa: mental health professionals’ opinion on willingness and role stress. Rev Colomb Psiquiatr.

[3] Takimoto Y (2022). Key components of the mental capacity assessment of patients with anorexia nervosa: a study of three countries. J Eat Disord.

[4] Gillett G. I eat therefore I am not. In: S Edition. editors. The Mind and its discontents. Oxford: Oxford University Press; 2009. p. 281–305.

[5] Asai K (2009). Are the functions of psychiatric review boards standardized?. J Jpn Assoc Psychiatr Hosp.

[6] Ramasamy RS (2021). Involuntary treatment of minors with severe and enduring anorexia nervosa. J Am Acad Psychiatry Law..

[7] Dyer C (2014). Anorexia patient should not be subject to any more compulsory treatment, court rules. BMJ.

[8] Silber TJ (2011). Treatment of anorexia nervosa against the patient’s will: ethical considerations. Adolesc Med State Art Rev.

[9] Hébert PC, Weingarten MA (1991). The ethics of forced feeding in anorexia nervosa. CMAJ.

[10] MacDonald C (2002). Treatment resistance in anorexia nervosa and the pervasiveness of ethics in clinical decision making. Can J Psychiatry Rev.

[11] Lewis P (1999). Feeding anorexic patients who refuse food. Med Law Rev.

[12] Guarda AS, Pinto AM, Coughlin JW, Hussain S, Haug NA, Heinberg LJ (2007). Perceived coercion and change in perceived need for admission in patients hospitalized for eating disorders. Am J Psychiatry.

[13] Osuji PI (2018). Relational autonomy in informed consent (RAIC) as an ethics of care approach to the concept of informed consent. Med Health Care Philos.

[14] Nakao-Hayashizaka KC (2022). End-of-life preparedness among Japanese Americans: a community survey. J Soc Work End Life Palliat Care.

[15] Ruhnke GW, Wilson SR, Akamatsu T, Kinoue T, Takashima Y, Goldstein MK (2000). Ethical decision making and patient autonomy: a comparison of physicians and patients in Japan and the United States. Chest.

[16] Elzakkers IF, Danner UN, Hoek HW, Schmidt U, van Elburg AA (2014). Compulsory treatment in anorexia nervosa: a review. Int J Eat Disord.

[17] Feiring E, Ugstad KN (2014). Interpretations of legal criteria for involuntary psychiatric admission: a qualitative analysis. BMC Health Serv Res.

[18] Bookman K, Zane RD (2020). Surviving a medical malpractice lawsuit. Emerg Med Clin North Am.

[19] O’Donoghue B, Lyne J, Hill M, Larkin C, Feeney L, O’Callaghan E (2011). Physical coercion, perceived pressures and procedural justice in the involuntary admission and future engagement with mental health services. Eur Psychiatr.

