# Supplementary material for: Indications for involuntary hospitalization for refusal of treatment in severe anorexia nervosa: a survey of physicians and mental health care review board members in Japan
Source: J Eat Disord. 2022 Nov 21;10:176. doi: 10.1186/s40337-022-00703-w (PMC9682757; doi:10.1186/s40337-022-00703-w)
Supplement: Supplementary file 1 — Additional file 1. Presentation of the cases and questions to the respondents. [file 40337_2022_703_MOESM1_ESM.docx]

**Additional file 1:**

**Title:** Presentation of the cases and questions to the respondents.

**Description:** The respondents were asked whether they would choose compulsory inpatient treatment or not for the following three cases. The questions posed to the physicians and committee members are also presented below.

CASE A:

Due to nutritional condition imposing a life-threat **(high risk of death due to malnutrition),** a **15-year-old** patient with an onset of anorexia nervosa **(developed 6 months prior with no previous treatment history at a medical institution)** comes to your hospital with their family members. However, despite the doctor’s advice, the patient continued to refuse both admission and outpatient treatment saying, “I do not want to receive any nutritional treatment, I do not want to get fat and I do not care even if my refusal leads to my death.” It might be possible to force treatment on him/her because he/she are not physically able to resist it due to them being weakened to the point where he/she have to stay in bed and cannot get up by themselves. There is no evidence that he/she have lost competence to judge.

CASE B:

Due to the nutritional condition being life-threatening **(high risk of death due to malnutrition),** a **20-year-old** patient with an onset of anorexia nervosa **(developed 6 months prior with no previous treatment history at a medical institution)** comes to your hospital with their family members. However, despite the doctor’s advice, the patient continued to refuse both admission and outpatient treatment saying, “I do not want to receive any nutritional treatment, I do not want to get fat and I do not care even if my refusal leads to my death.” It might be possible to force treatment on him/her because he/she are not physically able to resist it due to them being weakened to the point where he/she have to stay in bed and cannot get up by themselves. There is no evidence that they have lost competence to judge.

CASE C:

Due to the nutritional condition being life-threatening **(high risk of death due to malnutrition),** a **40-year-old** AN patient is in need of in-hospital treatment **(developed 24 years prior; has been brought to the hospital with the same condition several times but has refused admission each time)** was brought to your hospital by their family members. However, despite the doctor’s advice, the patient insists saying “I do not want to receive any nutritional treatment, I do not want to get fat and I do not care even if my refusal leads to my death." It might be possible to force treatment on them because they are not physically able to resist it due to him/her being weakened to the point where he/she have to stay in bed and cannot get up by themselves. There is no evidence that have lost competence to judge.

**Question for physicians**

QUESTION 1: Which decision do you make if the patient's family members wished for them to receive treatment?

QUESTION 2: Which decision do you make if the patient's family members respect the patient’s choice?

**Question for committee members**

QUESTION 1: Please select one option that you think is applicable to whether or not the patient is eligible for hospitalization based on the Mental Health and Welfare Law, if the patient's family members wished for them to receive treatment.

QUESTION 2: Please select one option that you think is applicable to whether or not the patient is eligible for hospitalization based on the Mental Health and Welfare Law, if the patient's family members respect the patient’s choice.
