# Supplementary material for: Indications for involuntary hospitalization for refusal of treatment in severe anorexia nervosa: a survey of physicians and mental health care review board members in Japan
Source: J Eat Disord. 2022 Nov 21;10:176. doi: 10.1186/s40337-022-00703-w (PMC9682757; doi:10.1186/s40337-022-00703-w)
Supplement: Supplementary file 2 — Additional file 2. Case to inquire about the presence or absence of mental capacity. [file 40337_2022_703_MOESM2_ESM.docx]

**Additional file 2**

**Title: Case to inquire about the presence or absence of mental capacity**

**Descriptoin:** This case was presented to the respondents to determine the mental capacity of the patient.

A patient with anorexia nervosa understands that he/she is considerably underweight and endangering his/her life by refusing treatment. He/she also still refuses hospitalization insisting that “I would rather be dead than gain weight, I do not want to be treated.” He/she seems to have no psychopathic symptoms, such as delusions or hallucinations.
